# Understanding cell signaling in cancer stem cells for targeted therapy – can phosphoproteomics help to reveal the secrets?

**DOI:** 10.1186/s12964-017-0166-1

**Published:** 2017-03-29

**Authors:** Wolfgang Gruber, Tamara Scheidt, Fritz Aberger, Christian G. Huber

**Affiliations:** Department of Molecular Biology, Cancer Cluster Salzburg, Paris-Lodron University of Salzburg, Hellbrunner Strasse 34, 5020 Salzburg, Austria

**Keywords:** Cancer stem cells, Phospho-signaling, Kinases, Phosphoproteomics, Tumor cell heterogeneity

## Abstract

**Background:**

Cancer represents heterogeneous and aberrantly proliferative manifestations composed of (epi)genetically and phenotypically distinct cells with a common clonal origin. Cancer stem cells (CSC) make up a rare subpopulation with the remarkable capacity to initiate, propagate and spread a malignant disease. Furthermore, CSC show increased therapy resistance, thereby contributing to disease relapse. Elimination of CSC, therefore, is a crucial aim to design efficacious treatments for long-term survival of cancer patients. In this article, we highlight the nature of CSC and propose that phosphoproteomics based on unbiased high-performance liquid chromatography-mass spectrometry provides a powerful tool to decipher the molecular CSC programs. Detailed knowledge about the regulation of signaling processes in CSC is a prerequisite for the development of patient-tailored multi-modal treatments including the elimination of rare CSC.

**Main body:**

Phosphorylation is a crucial post-translational modification regulating a plethora of both intra- and intercellular communication processes in normal and malignant cells. Small-molecule targeting of kinases has proven successful in the therapy, but the high rates of relapse and failure to stem malignant spread suggest that these kinase inhibitors largely spare CSC. Studying the kinetics of global phosphorylation patterns in an unbiased manner is, therefore, required to improve strategies and successful treatments within multi-modal therapeutic regimens by targeting the malignant behavior of CSC. The phosphoproteome comprises all phosphoproteins within a cell population that can be analyzed by phosphoproteomics, allowing the investigation of thousands of phosphorylation events. One major aspect is the perception of events underlying the activation and deactivation of kinases and phosphatases in oncogenic signaling pathways. Thus, not only can this tool be harnessed to better understand cellular processes such as those controlling CSC, but also applied to identify novel drug targets for targeted anti-CSC therapy.

**Conclusion:**

State-of-the-art phosphoproteomics approaches focusing on single cell analysis have the potential to better understand oncogenic signaling in heterogeneous cell populations including rare, yet highly malignant CSC. By eliminating the influence of heterogeneity of populations, single-cell studies will reveal novel insights also into the inter- and intratumoral communication processes controlling malignant CSC and disease progression, laying the basis for improved rational combination treatments.

## Background

Cancer is caused by the accumulation of genetic and epigenetic changes that eventually account for the unrestricted proliferative and metastatic capacity of malignant cells [1, 2]. Despite of having a common cellular and genetic ancestor, deep genome sequencing of cancer cells together with histopathological and molecular marker analyses revealed a surprising heterogeneity of cancer cells within the tumor mass. Following a Darwinian selection scheme, clonal evolution results in dynamic changes of subclones, which can account for disease progression and drug resistance in response to therapy [3–5]. Notably, the malignant capacity of clonal cancer cells differs considerably in terms of tumor initiation, propagation, metastatic spread and therapy resistance. In most - if not all malignancies - these highly aggressive traits can be ascribed to the presence of rare and self-renewing cancer cells. Since this rare subpopulation displays several stem-like cell characteristics and is likely to derive from long-lived tissue stem cells, these cells are commonly - but not exclusively - referred to as cancer stem cells [6, 7]. The terminology for self-renewing cancer cells with tumor initiating and maintaining properties is diverse, controversial, context-dependent and research-field specific. Here, we will use the term cancer stem cells (CSC) for rare self-renewing malignant tumor cells that have the ability to initiate, maintain and propagate heterogeneous malignancies (for details about the terminology and nomenclature of CSC see [8]).

## Cancer stem cells and tumor heterogeneity

**Fig. 1:**
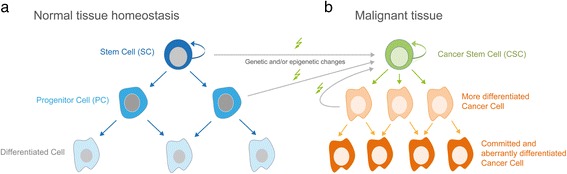
Scheme of the hierarchical stem cell model in healthy and malignant tissue. **a** Asymmetric cell division of a stem cell (SC; depicted as *dark blue* cells) in normal tissue results in the generation of a daughter stem cell as well as committed and dividing progenitor cells that can give rise to terminally differentiated cells (shown as *light blue* cells) of the given tissue. **b** Genetic and/or epigenetic alterations can transform stem cells and/or progenitor cells, leading to the escape from intracellular and extracellular control mechanisms that restrain aberrant cell proliferation and uncontrolled tissue growth. Constant self-renewal and the production of heterogeneous malignant progeny is considered a hall mark of cancer stem cells (CSC). The CSC model in malignant tissue represents a hierarchical organization, where rare self-renewing and long-lived CSC give rise to the tumor mass consisting of heterogeneous cancer cells with variable degree of differentiation and proliferative capacity (*orange* cells). CSC are more resistant to radiation- and chemotherapy calling for targeted approaches that eliminate CSC in multi-modal treatment strategies [134]

The hierarchical CSC model of malignant development and growth is a result of numerous recent genetic, cellular and molecular analyses of cancer heterogeneity (see below). However, the first evidence pointing to the existence of stem-like tumorigenic cells dates back at least several decades. Kleinsmith and Pierse demonstrated in 1964 that single embryonal carcinoma cells within a teratocarcinoma can give rise to multiple cellular lineages [9]. By performing 1700 single cell grafts, of which 43 formed teratocarcinomas composed of at least 14 different somatic tissues, this study provided experimental support for the stem cell theory of cancer. The basic concept of this model, however, has already been hypothesized in 1907 by Max Askanazy, a Prussian pathologist, who speculated that based on histological similarities between tumors and embryonic tissues, cancer arises from cells with properties similar to those of the early embryo [10]. Much has changed since then from both a technical and mechanistic point of view, but the basic concept of tumors arising from undifferentiated stem-like cells has recently been supported for many cancer entities, using sophisticated and state-of-the-art transplantation and genetic tools. Together, these seminal studies (for elaborate reviews see [6, 11, 12]) have led to a hierarchical rather than stochastic model of malignant development and growth driven by self-renewing cancer stem cells (Fig. 1).

The first evidence for clonal and stem cell-derived development of malignancies in man came from a study with patients suffering from chronic myeloid leukemia (CML). In 1967, Fialkow et al. investigated females heterozygous for the X-linked glucose-6-phosphate dehydrogenase (G-6-PD), resulting in the expression of only one of the two enzyme types in a single cell. By analyzing the blood cells of three female heterozygous G-6-PD patients, the team found exclusive expression of only one allele of G-6-PD in all CML cells of a patient, suggesting that the malignancy arose from a single hematopoietic stem cell [13].

Nearly 20 years later, the existence and phenotypic characterization of leukemia initiating CSC was reported by Bonnet and Dick for acute myeloid leukemia (AML) [14]. The authors found that only the rare CD34^+^ CD38^-^ subpopulation of undifferentiated leukemic cells possesses self-renewing and leukemia initiating capacity. Since this study was based on engraftment experiments in immunocompromised NOD/SCID mice, the leukemia initiating cells were termed SCID leukemia-initiating cells (SL-IC). Although the first concepts of the hierarchical CSC model were based on studies of leukemic malignancies (reviewed in [15]), multiple evidence has been provided since for the existence of CSC in numerous solid tumors. The first report of CSC in a solid malignancy came from studies of primary breast cancer samples. Al-Haji et al identified rare, undifferentiated CD44^+^/CD24^-/low^ cells as highly tumorigenic [16]. In this study, the authors demonstrated that as few as 100 CD44^+^/CD24^-/low^ cells were sufficient to initiate the growth of tumors that could be serially passaged, each time giving rise to heterogeneous tumors comprising rare self-renewing CD44^+^/CD24^-/low^ CSC and abundant non-tumorigenic cells.

During the past years, numerous reports have identified and confirmed the existence of rare CSC in the majority of human malignancies including cancers of the brain, the gastro-intestinal tract, skin and many other tissues [16–21]. Notably, CSC not only account for tumor initiation, growth and relapse in settings of minimal residual disease, dormancy and therapy resistance [22–25], but also are able to trans-differentiate for instance, into endothelial cells, thereby contributing to the tumor vasculature and malignant growth of glioblastoma [26]. As for the molecular determinants of CSC fate, it could be shown that the expression of a particular combination of transcription factors can reprogram non-CSC into CSC-like cells, analogous to the reprogramming and induction of pluripotent stem cells. In a glioblastoma model, expression of four factors, POU3F2, SOX2, SALL2 and OLIG2 in non-CSC is sufficient for the reprogramming of stem-like tumor-propagating cells (TPCs) with an epigenetic landscape comparable to the proper CSC population [27].

**Fig. 2:**
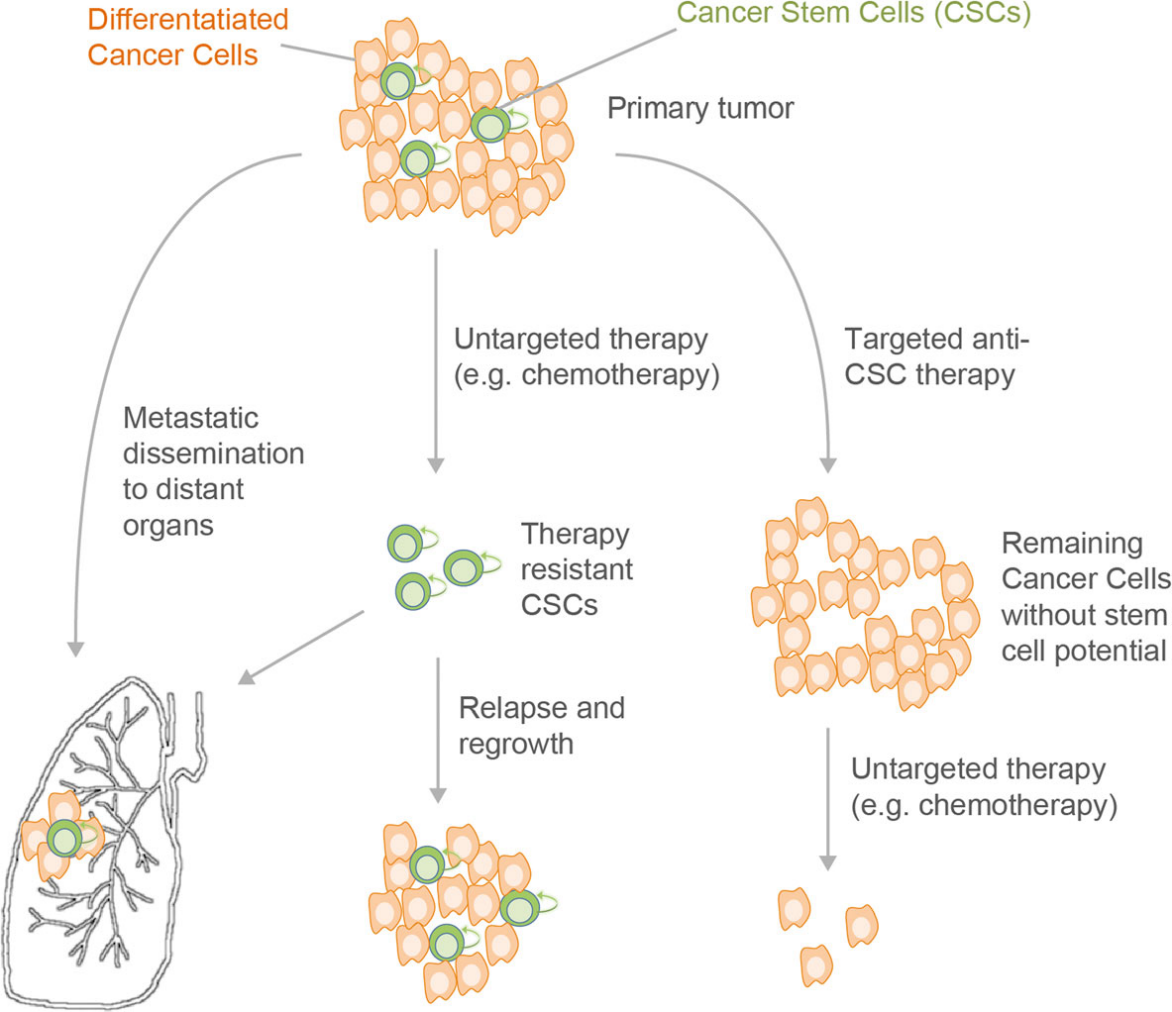
Cancer Stem Cells (CSC) display enhanced chemoresistance and account for metastases and disease relapse. A tumor typically consists of a minority of CSC, which give rise to more differentiated cancer cells. These differentiated tumor cells represent the majority of cells in the primary tumor, but have a limited self-renewal capacity. Untargeted therapy (e.g. chemotherapy) mainly affects highly proliferating non-CSC. Therapy resistant CSC are spared and can subsequently lead to tumor regrowth and therapy resistance in the initially responding patient (*middle panel*). Anti-CSC therapy prior to or together with untargeted therapy would hinder the tumors ability to regrow (*right panel*). Cancer cells with CSC properties can leave the primary tumor via blood or lymphatic vessels and form metastases in distant organs (*lower left panel*)

The notion that CSC are likely to derive from long-lived tissue stem cells has been intensely studied in transgenic mouse models suitable for genetic labeling of stem cells and lineage tracing of stem cell progeny in a defined genetic setting including selected cancer driver mutations (for review see [28]). Such studies revealed, for instance, rare Lgr5-positive intestinal crypt stem cells with hyperactive Wnt signaling as those cells that fuel the growth of intestinal adenomas. Like wild-type intestinal stem cells, Lgr5 positive adenoma stem cells reside in the bottom of the crypt niche, where they generate aberrantly proliferating Lgr5-negative adenoma cells that build the tumor mass [29, 30]. In line with a crucial role in fueling tumor growth, selective depletion of intestinal CSC resulted in rapid tumor regression, demonstrating the therapeutic potential of direct CSC targeting, although the relevance of these findings to human pathology and therapeutic relevance still remains to be addressed in detail [31] (for a general concept of CSC targeting see Fig. 2).

## Deciphering the phosphoproteome of CSC for the development of anti-CSC therapies

The highly malignant nature of CSC together with their pivotal role in disease relapse calls for a detailed and comprehensive understanding of the molecular processes regulating CSC behavior. Since kinases frequently represent the major effectors of oncogenic signals that can be efficiently targeted by small molecule drugs, we propose that the in-depth analysis of the phosphoproteome of CSC in combination with functional assays will allow the identification of kinases that determine the malignant phenotype of CSC. We consider this knowledge as essential prerequisite for the design of efficient combination treatments to eradicate CSC. If embedded in multimodal treatment regimens including immunotherapy, anti-CSC strategies are likely to significantly improve the overall survival of cancer patients by reducing malignant growth, metastatic spread, therapy resistance, and relapse rates.

The detailed and comprehensive analysis of rare CSC by -omics methods is a challenging endeavor, since CSC represent only a rare subpopulation of the tumor mass, posing severe constraints on the number of cells available for downstream investigations. The instrumental setup for the analysis of minute samples, therefore, has to be of sufficient sensitivity, particularly if it comes to technologies such as phosphoproteomics, where only a fraction of the respective protein molecules display post-translational phosphorylation marks. Aside from the technological challenges, the lack of universal and unambiguous CSC markers suitable for CSC isolation needs to be taken into account for the design of the isolation procedure.

Enrichment of rare CSC by their characteristic immunophenotype distinguishing CSC from non-CSC cells of the tumor bulk has been widely used and successfully applied. However, the choice and combination of surface epitopes is often specific only to a particular malignant entity and can result in the partial isolation of characteristic subpopulations of CSC [32, 33].

As an alternative, the increased activity of aldehyde dehydrogenase (ALDH) and certain efflux pumps in CSC allows to distinguish CSC from non-CSC. Increased ALDH activity can be translated biochemically into the generation of fluorescent signals. ALDH-positive cells can then readily be quantified and isolated by flow-cytometry and fluorescence-activated cell sorting, respectively. One of the first studies applying this strategy identified a rare ALDH-positive subpopulation of breast cancer cells with pronounced tumor-initiating potential consistent with ALDH-positive cells with CSC characteristics [34]. In addition, high level expression of ATP-binding cassette transporter proteins endows CSC with an efficient efflux detoxification machinery. Therefore, incubation of cancer cells with a cell permeable fluorescent dye such as HOECHST 33342 results in rapid and quantitative efflux of the dye in CSC while non-CSC retain a high intracellular concentration of HOECHST 33342. When analyzed by flow cytometry, CSC appear as dim population referred to as side population. Consistent with the dim side population being rich in CSC, HOECHST 33342 dim but not bright cells display high tumor initiating capacity [35–37].

CSC enrichment based on the differential immunophenotype or enzymatic activity of CSC and non-CSC is frequently applied and well established for a variety of cancer entities. However, none of these methods allows the selective expansion of CSC to readily increase CSC numbers to levels sufficient for unbiased global phosphoproteomics approaches. Compared to non-CSC, CSC have a much higher intrinsic capacity for clonal growth when cultured under specific in vitro conditions. For instance, growth of pancreatic cancer cells in 3-dimensional matrix cultures results in the formation of large, tumor-initiating spheres highly enriched for CSC [38, 39]. The clonogenic growth properties of CSC can therefore be used for the selective expansion of tumor-initiating CSC yielding cell numbers sufficient for elaborate phosphoproteomics studies.

## Phosphoproteome analysis of cancer and cancer stem cells

The role of protein phosphorylation in the control of cellular behavior has been well appreciated and intensely studied for many years. Phosphorylation serves as one of the most important post translational modifications (PTMs) of proteins to operate and reversibly control signaling [40]. Since phosphorylation is known to affect processes such as cellular growth, cell division, and metabolism, a dysfunction in protein phosphorylation can promote the development of various diseases such as cancer. Kinases catalyze the phosphorylation of serine, threonine or tyrosine residues within proteins using ATP as substrate. The requirement of precise control of kinase activity for the integrity of an entire tissue or even organism becomes evident by the fact that genetic alterations in kinase signaling pathways are frequently associated with the development and growth of cancer [41–44]. Therefore, a detailed and comprehensive knowledge of the phosphoproteome landscape of CSC is an important prerequisite for the design of efficient targeted therapies selectively blocking aberrantly active kinases and the malignant traits of CSC, respectively.

Phosphoproteome analysis or phosphoproteomics is a comprehensive technique analyzing the phosphoproteome of cells in a particular cellular state and biological context. The phosphoproteome comprises all phosphoproteins within a cell population or a single cell. According to Aebersold and Goodlett, phosphoproteomics tries to reveal the “trinity of protein phosphorylation analysis”, which is “identification of the site of phosphorylation, identification of the kinase responsible for the phosphorylation, and identification of the function and role of this phosphorylation” [45]. In the past, two-dimensional gel electrophoresis (2-DE) has been the dominant analysis technique for analyzing the phosphoproteome. 2-DE fractionates intact and undigested proteins by separation of the proteins by charge and molecular mass in two separate dimensions [46]. In particular, Phos-tag containing gels were developed, which enhance the separation of phosphoproteins through incorporation of Mn^2+^ or Zn^2+^ ions into the gel, for selective separation of phosphoproteins in SDS-PAGE gels. Followed by immunoblotting, a map of phosphorylated proteins can be created enabling the profiling of kinase activity in vitro [47].

While 2-DE has represented the golden standard for comprehensive proteome analysis for many years [48],the more generic nature of high-resolution tandem mass spectrometry coupled to one- or multidimensional high-performance liquid chromatography (HPLC-MS/MS) [49] meanwhile has superseded the 2-DE technique. In the so called “shotgun (phospho) proteomics” approach, extracted proteins of a cell population are first digested by a specific protease before being subjected to HPLC-MS/MS for separation and detection. The breakthrough in the technical development, which enabled the use of HPLC-MS/MS as a comprehensive revelation engine for proteins and peptides, was the invention of soft ionization techniques such as ESI (electrospray ionization) [50], which enable the direct mass spectrometric analysis of biological samples from liquid, often aqueous solutions. Nowadays, mass spectrometry is the primary identification and quantification tool for comprehensive phosphoproteomics [51, 52]. Moreover, identification technologies based upon the gas-phase fragmentation of peptide ions [53] and the matching of the resulting set of fragment ions with protein sequence databases [54–56] have laid the ground for the high-throughput identification and quantification of proteins in proteomic samples, enabling the analysis of more than 10,000 proteins in a single 12-day experiment [57].

## HPLC-MS/MS workflow for phosphoproteomics

A typical experimental design of a phosphoproteomics study first involves the isolation of the phosphoproteins, which is done by cell lysis in a lysis buffer assuring phosphatase and protease inhibition. After a complex sample preparation procedure of denaturation, reduction, and alkylation, the isolated proteins are digested into peptides. This is normally done by using proteases such as trypsin, chymotrypsin, or LysC, which provide peptides of a size highly suitable for mass spectrometric investigation [58]. Combinatorial approaches that complement trypsin by multiple proteases help to overcome the drawback of tryptic digestion, which often results in missing particular cleavage sites, particularly in the case of phosphorylation or other post translational modifications [59].

In contrast to the sample preparation in proteomics, the workflow for phosphoproteomics has to be expanded by procedures for phosphopeptide enrichment. Since the complexity of the cellular proteome hinders the direct analysis of phosphopeptides that are usually present in concentrations much lower than their non-phosphorylated analogues, further fractionation and phosphopeptide enrichment is needed to investigate the phosphoproteome. Different enrichment and fractionation methods have been applied, which were recently reviewed [51]. Typically, enrichment strategies rely on affinity chromatography taking advantage of the phosphate-specific binding abilities of certain metal oxides [60] (titanium dioxide, tin oxide [61]) or of immobilized metal ions such as Fe^3+^ [62] or Ga^3+^ [63]. The corresponding chromatographic modes have been termed metal oxide affinity chromatography (MOAC) or immobilized metal affinity chromatography (IMAC).

Since the detection of phosphorylated tyrosines is superimposed by the higher abundant serine and threonine phosphorylations in conventional shotgun phosphoproteomics approaches, immunoprecipitation based on phosphotyrosine antibodies has been implemented as an alternative enrichment strategy. Thus, the targeted enrichment of phosphorylated tyrosines prior to HPLC-MS detection improves the coverage of the phosphoproteome, especially when focusing on tyrosine phosphorylation by tyrosine kinases [51, 64, 65].

Furthermore, multidimensional chromatographic separations are usually applied for extensive fractionation of (phospho-) peptides [66]. Thereby the sample complexity is reduced and the instrument sensitivity is increased. Since peptides may contain both acidic and basic side chains, they can bear, depending on the pH of the solution, a positive or negative net charge, making them amenable to both cation- and anion-exchange chromatography [67]. Moreover, phosphorylation introduces a negative charge, thereby increasing the negative or decreasing the positive charge of a peptide, usually also resulting in a more hydrophilic nature of the phosphopeptides. Therefore, hydrophilic chromatographic separation techniques or combinations of charge-based/hydrophilic interaction modes are applicable [68].

**Fig. 3:**
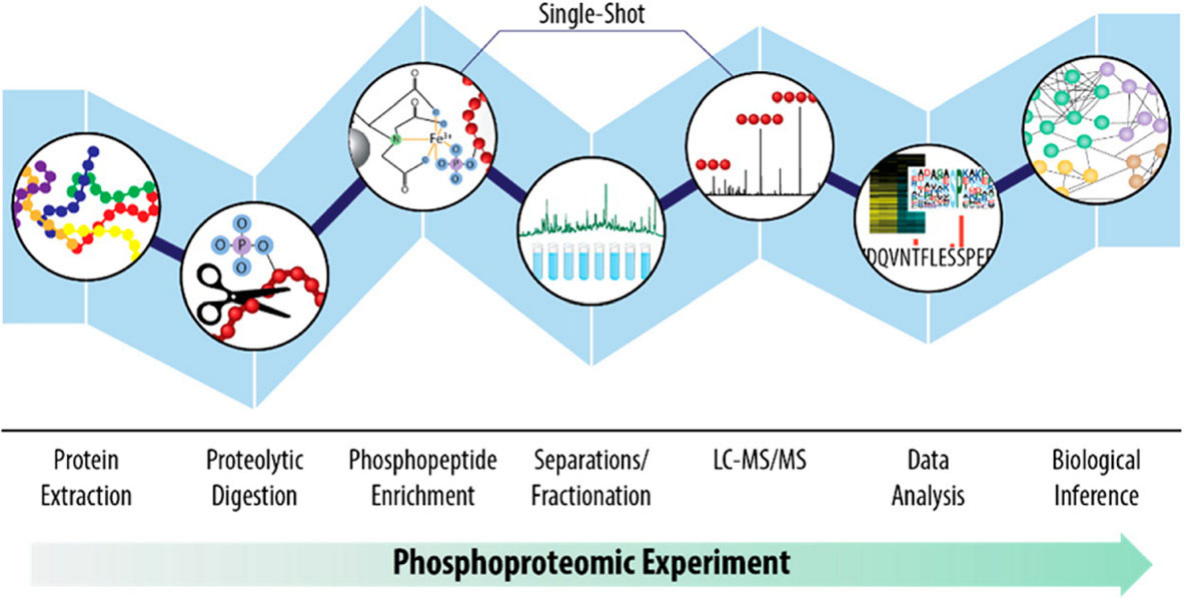
Typical phosphoproteomics workflow. Each step in a phosphoproteomic experiment can contribute to limitations in reproducibility and phosphoproteomic depth, which can ultimately restrict the biological insight obtained from an experiment. Concerted efforts in the phosphoproteomics community to improve each step in this workflow continue to advance our ability to sample the phosphoproteome with greater speed and depth, but comprehensive phosphoproteome coverage remains out of reach. Reproduced from [51] with permission of ACS Publications © 2015

The most commonly applied methods for separation in a first dimension are strong cation exchange chromatography (SCX) [69–71] or reversed-phase HPLC at high pH [72] besides electrophilic repulsion chromatography (ERLIC) [73] or hydrophilic interaction chromatography (HILIC) [74]. This first dimension is usually combined with a final (ion-pair) reversed-phase (IP-RP) separation before mass spectrometric detection via high-resolution mass spectrometry (HRMS) [51]. Offering the advantage of very high resolution and mass accuracy, high-resolution hybrid mass spectrometers such as quadrupole-time-of-flight (Q-TOF) [75], linear ion trap-Orbitrap (LTQ-Orbitrap), or quadrupole-Orbitrap (Q-Orbitrap) instruments [76] are the first choice in large-scale phosphoproteomics approaches. These instruments provide full scan spectra of intact peptides as well as fragment spectra of selected peptide precursor ions, which are then compared with databases for peptide identification by means of suitable computational tools [55, 56, 77]. Advantages and disadvantages of instruments have been reviewed elsewhere [51, 78]. A short summary of a typical phosphoproteomics workflow is shown in Fig. 3.

## Challenges of analyzing the phosphoproteome

Phosphoproteins and -peptides are bringing about special instrumental and sample preparation challenges. The availability of relatively high amounts of sample required for untargeted phosphoproteome analysis, typically in the range of 100 μg [68] to several milligrams of protein, may be problematic, especially when trying to analyze human material from biopsies [79]. This limitation may be overcome by employing targeted analysis by means of highly sensitive, mass spectrometry-based selected- or multiple reaction monitoring (SRM or MRM) methods [80]. Furthermore, Sequential Window Acquisition of all Theoretical Fragment Ion Mass Spectra (SWATH-MS) is evolving as a highly efficient global (phospho) proteome quantification strategy [51] and might facilitate the incorporation of tissue samples into SWATH-MS proteome maps similar to biobanks [81]. Microfluidic approaches to single-cell phosphoprotein analysis in a clinical context will be discussed in a separate section below.

Due to the sub-stoichiometric nature of protein phosphorylation, special sample preparation and phosphopeptide enrichment steps are required, as mentioned above. Besides, phosphopeptides provide significant difficulties for the mass spectrometric analysis [52, 82]. Phosphopeptides show lower ionization efficiencies in positive ionization mode due to ion suppression compared to non-phosphorylated peptides [83]. In addition, in the case of phosphoproteins, the labile phosphoryl group can be easily lost during fragmentation. This leads to an incident called neutral loss of 98 Da, which usually produces a dominant fragment ion and has to be considered for the identification of the peptides. Different fragmentation techniques have been applied and combined to improve the phosphopeptide identification such as collision-induced dissociation (CID) [84], higher-energy collision-induced dissociation (HCD), and electron-transfer dissociation (ETD) [85], but until now there is no universally applicable technique [86]. Furthermore, it is important to localize the phosphorylation to the corresponding amino acid residue. This phosphosite localization can be even more important and challenging than the peptide identification itself craving for an appropriate algorithm [87].

Moreover, tyrosine phosphorylation occurs 100–1000 times less than Ser/Thr phosphorylation, which requires phosphotyrosine-specific enrichment strategies as described above [51]. The study of tyrosine phosphorylation is important in unraveling signaling mechanisms connected to malignancies such as cancer, especially because the majority of FDA approved kinase inhibitors applied in tumor therapy target tyrosine kinases [88, 89].

In addition to the requirement of sophisticated and state-of-the-art technologies, also the dynamic nature of phosphorylation requires careful avoidance of enzymatic or chemical dephosphorylation by means of phosphatase inhibitors, making the analysis a challenging task [90]. Phosphorylation events are time dependent and thus phosphoproteomics can only provide a snapshot of the particular condition.

## Quantification of changes in phosphoproteome regulation

Quantification is essential to reveal changes in the phosphoproteome. It enlightens the proteins, which are significantly regulated in the particular experimental conditions in response to e.g. a defined treatment, and helps to resolve signaling networks. There are different quantification strategies applied for phosphoproteomic approaches mainly including isotope-labeling and label-free methods. The most dominant techniques focus on labeling of peptides or proteins mostly with stable isotopes, which can be readily distinguished by mass spectrometry [91]. Stable isotope labeling by amino acids in cell culture (SILAC) is a very common metabolic in vivo labeling method before proteolytic digestion [92]. Thereby, during protein biosynthesis the cells incorporate isotope-labeled amino acids provided in the cell culture medium or in the feed for animal models.

Peptides can also be isotope-labeled during the tryptic digestion of proteins through incorporation of heavy oxygen from H_2_^18^O. Moreover, reductive dimethylation labeling using regular or deuterium-labeled forms of formaldehyde and sodium cyanoborohydride is an efficient post digestion labeling method applied for full MS quantification by comparing extracted ion chromatogram peak areas corresponding to the differently isotope-labeled peptide species [93]. Isotope-labeling of proteins or peptides can also be performed upon chemical derivatization with isotope-labeled, mostly amino- or thiol-reactive agents such as Isotope-coded Affinity Tags (ICAT) [94] or Isotope-Coded Protein Labels (ICPL) [95].

Finally, tags like isobaric tags for relative and absolute quantitation (iTRAQ) [96], or tandem mass tags (TMT) [97] can be used to quantify phosphopeptides by tandem mass spectrometry [98]. Here, quantification is enabled by tandem mass spectrometry (MS/MS) after fragmentation of the phosphopeptide upon generating reporter ions to obtain ratios between controls and treatments. One of the major advantages of isobaric labeling is the economization of measurement time and expense by merging of multiple (up to ten) samples. A more time consuming but attractive method for quantitative phosphoproteomics is label-free quantification of peptide signals in independent HPLC-MS/MS analyses [99, 100]. This is especially interesting for phosphoproteomics, since it does not require any further labeling and thus saves costs and prevents interferences with the phosphate group of the peptides. Nevertheless, there is a strong requirement for careful experimental design and/or normalization strategies in order to obtain comparable signal intensities [101].

## Phosphoproteomic applications for the analysis of cancer cells

HPLC-MS/MS based phosphoproteomics represents a discovery driven approach, which can help to track new drug targets and illuminate up- and downstream signaling molecules. Furthermore, phosphoproteomics can help to give new insights into phosphorylation networks and kinase-substrate interactions.

The human epidermal growth factor (HER) family of receptor tyrosine kinases was one of the first targets, which was addressed by phosphoproteomic analysis. The first studies analyzed changes in phosphorylation focusing on the analysis of phosphoproteins after enrichment by phosphotyrosine antibodies to examine the effect of epidermal growth factor (EGF) stimulation [102]. Although these studies provided insight into activation profiles of key proteins involved in epidermal growth factor receptor (EGFR) signaling and other unknown downstream proteins, they lack a global view to the phosphoproteome.

One of the first large-scale analyses of tyrosine kinase activity in lung cancer was performed in 2007 by Rikova et al., who identified 50 tyrosine kinases and over 2500 downstream substrates [103]. They confirmed well-known tyrosine kinases involved in oncogenic signaling such as EGFR and hepatocyte growth factor receptor (HGFR or c-Met). Furthermore, it was shown that activated forms of anaplastic lymphoma kinase (ALK) and receptor tyrosine kinase (ROS) can be identified in lung cancer cells, in particular in non-small cell lung cancer cell lines (NSCLC). A first deep and extensive view of tyrosine kinase activity and downstream signaling networks was described.

Revealing phosphoproteomic dynamics becomes more and more important especially in the area of cancer research. The first study elucidating temporal dynamics of phosphorylation upon growth factor stimulation was performed by Olsen and Mann in 2006. According to their discoveries, EGF-signaling is regulated by phosphorylation of a variety of transcriptional regulators, amongst others signal transducer and activator of transcription 5 (STAT5), transcription factor MYC, and transcription factor JUND, within a short time frame of 20 min. By following regulatory changes over a particular time frame, signaling outcomes could be connected to responsible upstream or downstream events [104].

Quantitative phosphoproteomic profiling was already used to portrait different tumorigenic signaling pathways, to compare different tumor entities and to analyze the heterogeneity of tumors. Only recently Schweppe et al. applied a Super-SILAC approach for decoding global phospho-signaling networks in NSCLC patient samples. They were able to differentiate between different types of non-small cell lung cancer populations due to changes in particular oncogenic drivers such as epidermal growth factor receptor 2 (ErbB2) and RAF/MEK/ERK signaling [105]. The RAF/MEK/ERK signaling is important for cellular growth, malignant transformation and drug resistance [106]. The regulation of stromal cells by oncogenic KRAS (Kirsten rat sarcoma viral oncogene homolog) in pancreatic ductal adenocarcinoma (PDA) cells was demonstrated by Tape et al. [107]. They performed an innovative sample preparation method called automated phosphopeptide enrichment (APE), where magnetic TiO_2_ and Ti-IMAC microspheres are used to enrich phosphopeptides by employing a magnetic particle handling robot [108]. They investigated the cell-autonomous and non-cell autonomous signaling effects of oncogenic KRAS on the phosphoproteome of PDA. Thereby, a cell-autonomous activation of ERK 1/2 was determined resulting in an induction of Map kinase and cyclin dependent kinase motifs. Likewise, oncogenic KRAS was demonstrated in a quantitative proteomic analysis to control PDA cells by influencing the Sonic Hedgehog (SHH)-Smoothened (SMO)-GLI axis of stromal cells. The stromal-driven tumor cell phosphoproteome moreover differed from the oncogenic KRAS regulated cell-autonomous phosphoproteome revealing reciprocal signaling of the stromal cells. This evidence emphasizes the importance of focusing on tumor heterogeneity in cancer studies and therapy.

Phosphoproteomics and proteogenomics can help to understand mechanisms of resistance to cancer therapeutics and predict efficacy or adverse reactions relevant for personalized medicine. As a comprehensive technique, phosphoproteomics offers the opportunity to study changes in the phosphorylation of targeted proteins after treatment and thus can be used as an investigation tool for pre-clinical and clinical investigations. Thereby it can be used to improve and expand current drug treatment systems [105] by tailoring medication for therapy to individual responsiveness and tendency for side effects. By applying phosphoproteomics on metastatic castration-resistant prostate cancer (CRPC) material, Drake and colleagues could identify phosphorylation of key mediators in six major signaling pathways, including the cell-cycle pathway, DNA repair pathway, AKT/mTOR/MAPK pathway, and the nuclear receptor pathway, which revealed potentially useful information for patient stratification and targeted therapy [109].

Proteogenomics parses the relation of genetic alterations to functional protein expression by comparison and integration of RNA and DNA sequencing data and (phospho) proteomics to infer their particular influence on the resulting phenotype [110, 111]. In breast cancer, the analysis of the phosphoproteome identified several phosphorylated kinases and a G Protein-coupled receptor cluster that could not be detected at the mRNA level [110]. Previous proteogenomic characterization of high-grade serous carcinoma (HGSC), which comprises the majority of ovarian cancer cases, included phosphopeptide analysis and demonstrated the added value of protein phosphorylation data when correlating pathway activity with patient survival [112]. Another proteogenomic study characterized rectal cancer patients and used proteomics data to prioritize candidate driver genes [111].

**Fig. 4:**
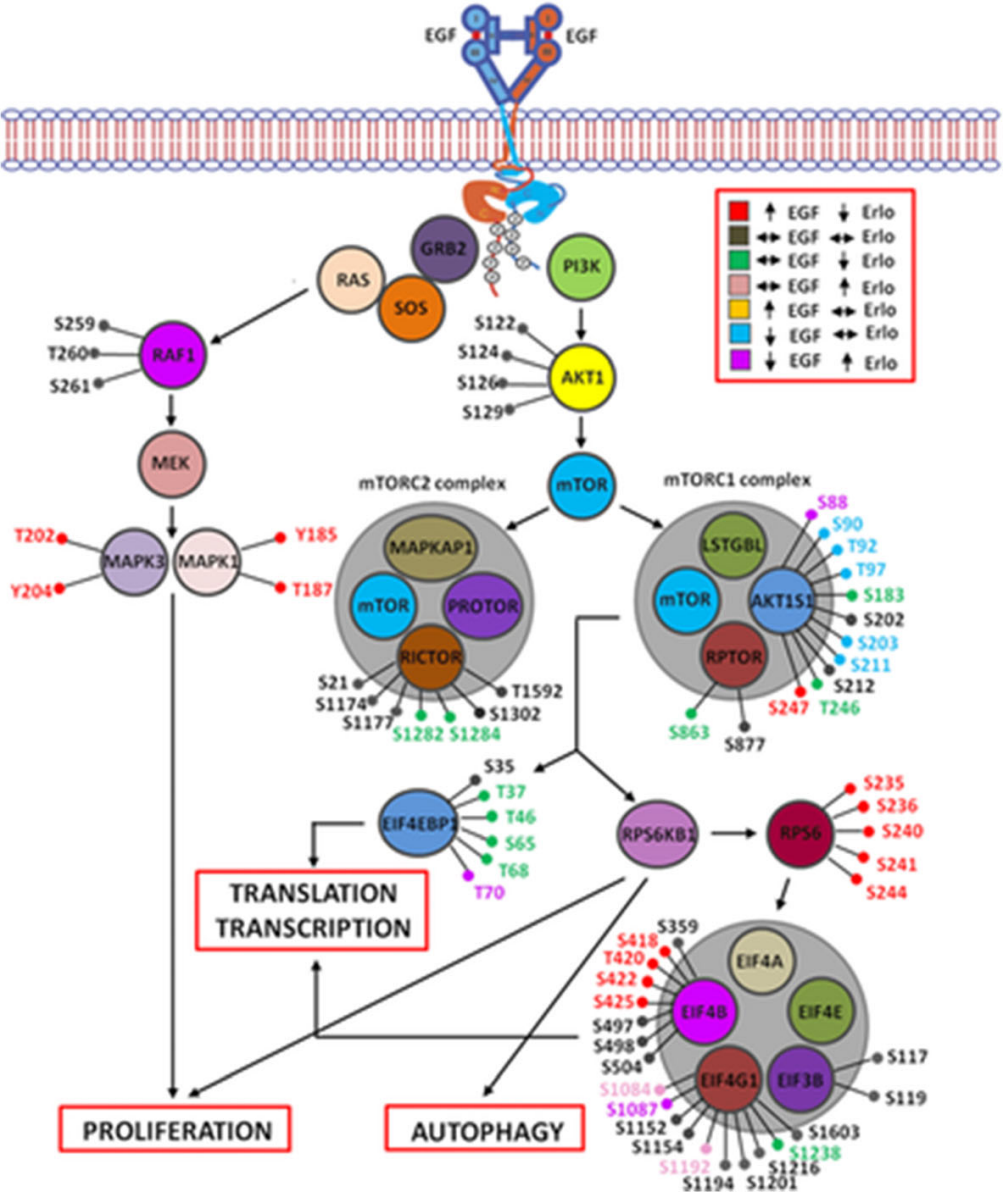
Phospho-sites identified in proteins of the RAS-RAF-MAPK and PI3K-AKT signaling pathway in a lung adenocarcinoma cell line harboring a L858R mutation in the kinase domain of EGFR, which is reactive to treatment with erlotinib. Reproduced from [113] with permission from Wiley-VCH © 2015

In the past decade, the focus has shifted towards the functional and temporal analysis of changes within particular oncogenic pathways upon treatment with tyrosine kinase inhibitors as potent cytostatic drugs for the treatment of various cancers. Zhang et al. examined the global phosphoproteome after erlotinib treatment, a tyrosine kinase inhibitor for the treatment of lung cancer. They utilized lung adenocarcinoma cell lines harboring mutations in the kinase domain of EGFR, making them either sensitive or resistant to erlotinib treatment. They compared phosphorylation events and canonical pathways enriched in the sensitive or resistant cells [113]. Particular differences in EGFR connected pathways and changes in the phosphorylation patterns of regulatory proteins such as phosphorylated AKT (pAKT) and pERK (phospho-extracellular-signal regulated kinase) depending on erlotinib treatment of the resistant or sensitive cells were observed (Fig. 4). Their study gives novel impressions of phosphorylation events affected by erlotinib treatment and provides insights into possible mechanisms of drug resistance.

Only recently Wu et al. identified Focal Adhesion Kinase 2 as a modulator of tamoxifen resistance in breast cancer. They treated MCF7 breast cancer cells for 6 months with the selective estrogen-receptor modulator tamoxifen or ethanol as a vehicle control in vitro. SILAC was used to perform quantitative phosphoproteomic profiling based on HPLC-HRMS. By systematically analyzing the 2189 identified phosphorylated proteins, the focal adhesion pathway was identified as one of the most enriched signaling pathways. Protein phosphorylation was significantly elevated in the tamoxifen resistant cells. The 27 hyperphosphorylated proteins included the focal adhesion kinases FAK1 and FAK2 in the tamoxifen resistant breast cancer cells. In ongoing investigations by using real-time PCR, Western blot analyses, and immunofluorescence staining the overexpression of FAK2 in tamoxifen resistant cells was confirmed. Finally, siRNA knockdown of FAK2 significantly reduced the proliferation of the MCF7-tamoxifen resistant cells and thus confirmed the pivotal role of FAK2 for tamoxifen resistance in these cells [114].

## CSC – an intricate challenge for proteomic and phosphoproteomic profiling

CSC are of main interest both for biomedical research and clinical therapy. As it has been introduced above, CSC account for metastasis, relapse, and resistance to cancer therapeutics in different cancer entities. Analyzing CSC remains a challenge due to their low abundance and the task to specifically isolate these cells (see above).

Since phosphorylation patterns and dynamics are crucial for the regulation of normal and malignant cellular behavior, future studies are to focus on phosphoproteomics to investigate cancer stem cell signaling. Proteomic profiling has already been applied to different cancer stem cell entities. In 2010, one of the first quantitative profiling studies of pancreatic CSC was published by Dai et al. They solved the problem of the limited number of CSC gained from xenograft mouse models of primary human pancreatic adenocarcinomas by applying a two-dimensional approach [115] that combined capillary isoelectric focusing and fraction collection in combination with nano reversed-phase HPLC-MS/MS followed by label-free quantification [115]. With this approach, they identified mitochondrial dysfunction as the top regulated pathway in the CSC population compared with the bulk tumor group. Moreover, other pathways known to be involved in cellular growth and proliferation such as VEGF signaling were shown to be enriched in CSC. Also, Interleukin signaling, Ras homologue gene family member A (RhoA), and integrin signaling pertaining to inflammatory and immunological pathways were found to be associated with CSC communication. Their results underline the connection between inflammation and carcinogenesis.

Recently, the proteome of sonic hedgehog driven human medulloblastoma stem-like cells was analyzed before and after retinoic acid differentiation [116]. The stem-like cells isolated from human infant medulloblastoma samples were further cultured as neurospheres in selective medium. HRMS following HPLC separation determined heat shock protein 70 as overexpressed in stem-like cells. Furthermore, the nuclear factor kappa-light-chain-enhancer of activated B-cells (NF-κB) complex and tumor suppressor protein p53 were illuminated as pivotal players for cancer and stemness networks. Ongoing investigations showed that the phosphorylated p65 subunit of the NF-κB complex was highly expressed in these cancer stem cells, thereby identifying new key biological players involved in cancer stem cell biology of medulloblastoma.

To better understand dynamic signaling processes in CSC, Nilsson et al. initiated the first quantitative phosphoproteomic analysis of glioblastoma stem cells in 2010. They scrutinized glioblastoma stem cells (GSC) derived from human tumors and cultured them as neurospheres. These cells were treated with the novel JAK2/STAT3 phosphorylation inhibitor WP1193 and/or the JAK/STAT3 activator IL-6 under normoxic and hypoxic conditions [117]. Six different conditions were compared by using TMT labeling prior to HILIC fractionation and TiO_2_ enrichment. The separation was performed by RP-HPLC and detection by HRMS resulting in a total of 3414 proteins detected. Subsequent data evaluation linked 21 highly regulated proteins to STAT3, HIF1α (hypoxia inducible factor 1 alpha) and IL-6 signaling.

Several phosphoproteins linked to metabolic changes were observed under hypoxic conditions besides 11 proteins connected to HIF1α. Mitogen-activated protein kinase 1 (MAPK1)-expression in particular was increased reflecting HIF1α activation. Comparing normoxic and hypoxic conditions, they showed that hypoxic GSC were less responsive and thus more resistant to treatment with WP1193. Under treatment with WP1193 in combination with IL-6 they observed increased Insulin-like growth factor I (IGF1) signaling in both normoxic and hypoxic cells which confirmed the modulatory role of IGF1 in glioblastoma proliferation and migration [118]. Even though the effect of hypoxia on glioblastoma growth was well described based on their data, this study did not focus on the analysis of phosphorylation sites and kinase substrate interactions. Thus, they could not enlighten the deeper effect of different treatment conditions to the phosphorylation dynamics in glioblastoma stem cells.

Kozuka-Hata et al. addressed glioblastoma initiating cells two years later by investigating the effect of EGF stimulation on initiating cells from glioblastoma patients [119]. They used SILAC for quantification and TiO_2_ columns to enrich the phosphopeptides prior to HPLC-MS/MS analysis. By searching against a human RNA database, they identified a novel peptide encoded by supervilin-like (LOC645954), which showed altered phosphorylation patterns upon EGF stimulation in a cell-type dependent manner. They started to look deeper into phosphorylation sites and their influence on communication and regulation of GSC. Out of 6073 phosphopeptides encoding 2282 phosphoproteins, 635 proteins belonging to the ErbB and mTOR signaling were shown to be upregulated in these CSC.

Still, our understanding of CSC regulation via phosphorylation remains largely incomplete. Only recently, the downstream signaling of stromal cell-derived factor 1 (SDF-1)/G protein-coupled receptor chemokine receptor 4 (CXCR4) in breast CSC has been examined [120]. The critical role of CXCR4 for tumor progression has already been known from O’Hayre et al., who examined the CXCL12/CXCR4 signaling network in chronic lymphatic leukemia (CLL) in 2010 but due to technical limitations, this work lacked comprehensive phosphosite analysis [121]. Yi et al. isolated CD44^high^/CD24^low^ CSC from human mammary epithelial cancer cells (HMLER) and cultured them as tumor spheres. Phosphorylation events induced by 10 min treatment with SDF-1 with or without transient CXCR4 knockdown were compared. Phosphorylation changes were observed in several proteins with cell regulatory functions such as GTPase activating proteins and histone modification enzymes.

Furthermore, they more deeply analyzed phosphorylation-affected kinases and phosphatases, among them ERK1 and serine/threonine-protein kinase 4 (PAK4), which were already known to be involved in the SDF-1/CXCR4 signaling cascade. PAK4 was already described as being important for the development of breast cancer [122]. Besides, 44 kinases out of 50 at least 2-fold elevated kinases detected have been not known to be related to this signaling machinery before. Furthermore, 70 phosphosites of the 87 phosphosites detected in these kinases were still undiscovered. By examining kinase-substrates and phosphatase-substrates of 266 phosphoproteins with increased phosphorylation, multiple upstream kinases were found to be mediated by SDF-1/CXCR4 signaling. These were upstream kinases such as Pyruvate dehydrogenase kinase 1 (PDK-1), ERK1, GSK3 β for 5 phosphoproteins such as PKA (protein kinase A) and NF-κB. Moreover, a MAPK network downstream of SDF-1/CXCR4 signaling could be created providing novel insights into the resulting system-wide phosphorylation dynamics [120].

In spite of a remarkable progress over the decades made in the field of CSC research, analyzing the global phosphoproteome and phosphorylation dynamics of this subpopulation of cells is still not routinely practicable. CSC expansion by cell cultivation is mostly needed to obtain enough material for the analysis, which, however, can distort a realistic situation and reduce the clinical relevance.

## Single cell proteomics for CSC investigation

One of the major disadvantages of current phosphoproteomics approaches is the need for relatively large amounts of cells samples, i.e. in the range of several million cells. This inevitably results in the study of heterogeneous cell populations, where the protein amount of each single cell and the respective phosphorylation pattern may vary considerably. Variability in phosphorylation-dependent signaling can influence the phenotype and quality of tumors, indeed it can be a reason for the formation of CSC [123]. CSC and bulk cancer cells are known to show inter- and intratumoral heterogeneity with marked differences in their malignant capacities. This versatility of a cancer (stem) cell population can be influenced by the microenvironment and/or intratumoral communication processes that induce different cell specific gene expression states [124] (for reviews see [3, 125]).

**Fig. 5:**
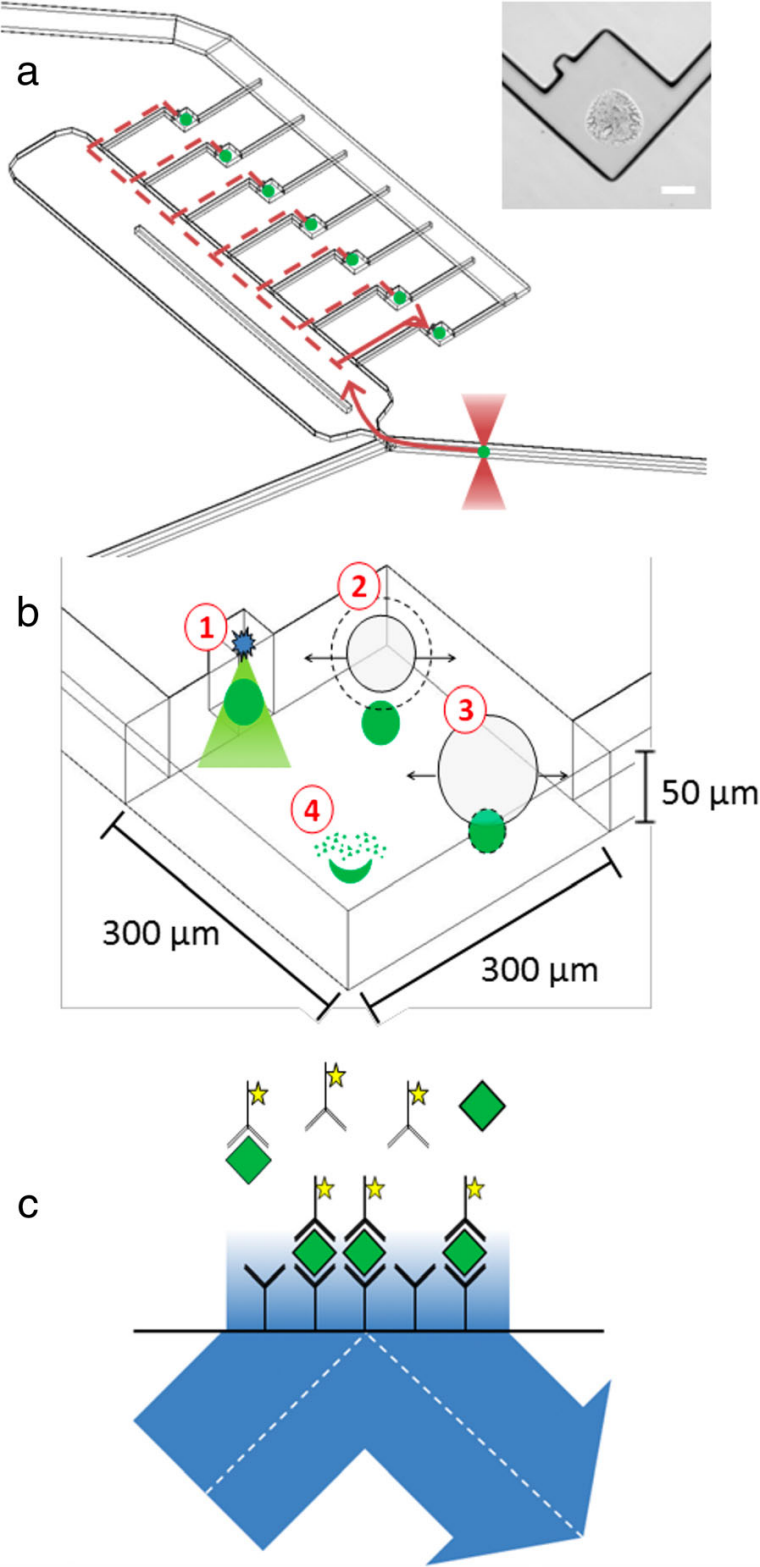
Microfluidic approach to single-cell phosphoproteomics. **a** The optical trap is used to move cells (*green circles*) from flow to analysis chambers. Inset: bright-field image of an antibody spot aligned within a chamber. Scale bar = 100 μm. **b** Single cells (*green circles*) are lysed by the delivery of a single 6 ns pulse at λ = 1064 nm 10 μm above the center of the cell. (1) At sufficient irradiance the medium breaks down to form a localized plasma; (2) An outwardly propagating shockwave and an expanding cavitation bubble are produced; (3) the cell is lysed due to shear stress from the expanding cavitation bubble; and (4) cellular constituents are released into the chamber. **c** Single cell protein levels are measured using an antibody spot. Chamber volume is 4.6 nL and results in favorable kinetics. By employing TIRF, only 10 fluorophores within 200 nm of the surface are imaged, which are assumed to be antibody/antigen bound. Reproduced from [129] with permission from the Royal Society of Chemistry © 2011

Until now, there are still technical limitations to perform phosphoproteomics at the single-cell-level, with sensitivity being the primary constraint [90]. For a comprehensive state-of-the-art phosphoproteomics approach the protein amount of a single cell is too low. Thus, the innovative approaches are based on the implementation of microfluidic systems in combination with very sensitive detection schemes of phosphoproteomics. In particular, lab-on-a-chip technologies should enable and simplify single-cell phosphoproteomic analyses [126]. Wei et. al only recently reported the first single-cell phosphoproteomics approach to study signaling dynamics in glioblastoma with a focus on development of drug resistance. They used the single-cell barcode chip technology (SCBC) to investigate more than a dozen of proteins and phosphoproteins [127, 128]. In this setup, one-cell microchambers were used to isolate single cells as illustrated in Fig. 5 [129]. These microchambers were connected by programmable valves to lysis buffer-containing storage cavities, such that on-chip cell lysis could be performed. Each microchamber could be covered with a chip that featured an antibody-barcoded stripe which was used to capture the released (phospho) proteins. Detection of the (phospho)proteins was subsequently done by fluorescently labeled secondary antibodies [130, 131]. Thereby activation of ERK- and proto-oncogene tyrosine-protein kinase Src signaling was detectable and linked to the cause of resistance to CC214-2 – an mTOR kinase inhibitor [132].

However, this approach is far from being comprehensive and unbiased. Antibody arrays are used to capture and quantify the proteins and phosphoproteins of interest. The barcode protein assay exhibited comparable dynamic ranges to commercially available ELISAs for around 12 proteins [127]. Meanwhile the number of detectable proteins was extended up to around 40 proteins per cell. Nevertheless, this targeted concept using prior knowledge about the tumor can hardly be compared with the discovery-driven process of unbiased HPLC-MS/MS based phosphoproteomics. Nevertheless, it can be feasible for implementation into the clinics, since only small amounts of material are needed and assays can be customized easily. There have been many attempts of combining this microfluidic principle with mass spectrometry, then called Chip-MS (for a review see [133]). These techniques are still in progress to be automated and improved but they combine both advantages of the downscaling feature of the microchip and the sensitive and discriminative detection capabilities of the MS instrument.

## Conclusions

The highly malignant nature of rare CSC such as their exquisite capacity to initiate and fuel tumor growth, to seed metastases and their pronounced intrinsic resistance to chemo- and radiation therapy – a frequent cause for patients´ relapse - calls for efforts to decipher the malignant code of the phosphoproteome. Understanding the complex phospho-signaling landscape of CSC will support the development of innovative multi-modal treatments including small-molecule targeting of key CSC kinases in combination with for instance, immunotherapy to significantly improve the overall long-term survival of patients.

Experimental workflows offering sufficient sensitivity and extensiveness for unbiased phosphoproteome analysis represent a real challenge in the investigation of signaling in heterogeneous populations of tumor cells. Nevertheless, in the past two decades, significant improvements in the detection techniques in terms of detection limits and structural information have enabled phosphoproteomic studies with very low amounts of sample down to the single-cell level. Moreover, the dynamic nature of phosphorylation itself provides challenges from the biological system, requiring very rapid quenching and sample preparation pipelines. Examining the phosphorylation events at single cell level is a desirable approach, but currently is restricted to the pre-selection of candidate phosphoproteins.

Comprehensive HPLC-MS/MS phosphoproteomics based on the analysis of single CSC represents an innovative and illuminative approach to investigate tumor initiating cells in great detail. In the future, with customized, enhanced and improved instrumentation this technique will likely become a routine part of modern clinical diagnosis and analysis as well as an essential method in the area of precision oncology.

## Abbreviations

2-DE: Two-dimensional gel electrophoresis
ALDH: Aldehyde dehydrogenase
ALK: Anaplastic lymphoma kinase
AML: Acute myeloid leukemia
APE: Automated phosphopeptide enrichment
ATP: Adenosine triphosphate
CID: Collision-induced dissociation
CLL: Chronic lymphatic leukemia
CML: Chronic myeloid leukemia
CRPC: Castration-resistant prostate cancer
CSC: Cancer stem cells
CXCR4: G protein-coupled receptor chemokine receptor 4
EGF: Epidermal growth factor
EGFR: Epidermal growth factor receptor
ErbB: Epidermal growth factor receptor
ERLIC: Electrophilic repulsion chromatography
ESI: Electrospray ionization
ETD: Electron-transfer dissociation
FAK1/2: Focal adhesion kinase 1/2
G-6-PD: Glucose-6-phosphate dehydrogenase
GLI: Glioma associated oncogene
GSCs: Glioblastoma stem-like cells
GSK3 β: Glycogen synthase kinase 3 β
HCD: Higher-energy collision-induced dissociation
hCNS-SC: Human central nervous system stem cells
HER: Human epidermal growth factor receptor
HGFR: Hepatocyte growth factor receptor
HGSC: High-grade serous carcinoma
HIF1α: Hypoxia inducible factor 1 alpha
HILIC: Hydrophilic interaction chromatography
HMLER: Human mammary epithelial cancer cells
HPLC: High-performance liquid chromatography
HRMS: High resolution mass spectrometry
HSP: Hematopoietic stem and progenitor cells
ICAT: Isotope-coded Affinity Tags
ICPL: Isotope-coded Protein Labels
IGF1: Insulin-like growth factor I
IMAC: Immobilized metal affinity chromatography
IP-RP: Ion-pair reversed phase
iTRAQ: Isobaric tags for relative and absolute quantitation
JUND: Transcription factor Jun-D
KRAS: Kirsten rat sarcoma viral oncogene homolog
Lgr5: Leucine-rich repeat containing G protein-coupled receptor 5
MAPK1: Mitogen-activated protein kinase 1
MOAC: Metal oxide affinity chromatography
MRM: Multiple reaction monitoring
MS: Mass spectrometry
MS/MS: Tandem mass spectrometry
NFκB: Kappa-light-chain-enhancer of activated B-cells
NSCLC: Non-small cell lung cancer
PAK4: Serine/threonine-protein kinase 4
pAKT: Phosphorylated protein kinase B
PDA: Pancreatic ductal adenocarcinoma
pERK: Phospho-extracellular-signal regulated kinase
PKA: Protein kinase A
PTM: Post translational modification
Q-TOF: Quadrupole-time-of-flight
RhoA: Ras homologue gene family member A
ROS: Receptor tyrosine kinase
SCX: Strong cation exchange chromatography
SDF-1: Stromal cell-derived factor 1
SDS-PAGE: Sodium dodecyl sulfate polyacrylamide gel electrophoresis
SHH: Sonic hedgehog
SILAC: Stable-isotope labeling by amino acids in cell culture
SL-IC: SCID leukemia-initiating cells
SMO: Smoothened
Src: Proto-oncogene tyrosine-protein kinase
SRM: Selected reaction monitoring
STAT5: Signal transducer and activator of transcription 5
SWATH-MS: Sequential window acquisition of all theoretical fragment ion mass spectra
TMT: Tandem mass tags
TPCs: Stem-like tumor-propagating cells
